# S201. RELATION BETWEEN PSYCHOTROPIC DRUGS AND SEIZURE THRESHOLD IN ELECTROCONVULSIVE THERAPY

**DOI:** 10.1093/schbul/sby018.988

**Published:** 2018-04-01

**Authors:** Seung Hyun Kim, Jung-Seo Yi, Su-Hyuk Chi, Hyun-Ghang Jeong

**Affiliations:** 1 Korea University, Guro Hospital College of Medicine; 2 Hallym University College of Medicine, Kangnam Sacred Heart Hospital; 3 Korea University, Guro Hospital

## Abstract

**Background:**

Electroconvulsive therapy (ECT) is the most popular way to stimulate brain for achieving therapeutic effects. The therapeutic effect of ECT results from the induction of a generalized seizure. The minimal amount of electrical energy needed to induce seizures is known as the seizure threshold (ST). It is commonly believed that treatment efficacy is related to stimulus dose relative to ST, but higher stimuli usually also increase unwanted side effects. Therefore, ST is an important issue in conducting ECT. Most patients including schizophrenics undergoing ECT take concomitant psychotropic drugs, but little information is available on how these drugs affect ST. Our study aimed to analyze the relationship between ST and psychotropic drugs in patients treated with ECT.

**Methods:**

We retrospectively reviewed the medical charts of 43 patients who received ECT at Korea University Guro Hospital between February 2009 and June 2015. Patients with a history of seizure disorders or other medical emergent conditions were excluded. A total of fifty-eight subjects received ECT during the study period. Patients were excluded if treatment was aborted due to side effects or any other reasons before the 10th session (n=12) because we intended to investigate the ST shift during the course of consecutive ECT sessions. We included 43 subjects in the final data analysis. Patients’ psychiatric disorders were diagnosed according to the Diagnostic and Statistical Manual of Mental Disorders (DSM-IV-R) by at least two experienced psychiatrists. ECT was administrated with concurrent antipsychotics and antidepressants.67.4 percent of subjects were diagnosed as schizophrenia and 20.9 percent of subjects were diagnosed as major depressive disorder. We used stepwise multivariate correlation analyses for examining the associations between ST and psychotropic drugs. Data are presented as initial ST, the difference in ST between the first and 10th sessions (ΔST10th), and the mean difference in ST between the first and last sessions (mean ΔSTlast). We used chlorpromazine-equivalent dose for antipsychotics and fluoxetine-equivalent dose for antidepressants.

**Results:**

Of the 43 patients included in the study, 20 were male, and the other 23 were female. The mean age of all participants was 41.44 years (SD=15.89). Patients were taking the following antipsychotics and antidepressants: clozapine (n=6), amisulpride (n=9), aripiprazole (n=5), olanzapine (n=18), risperidone (n=1), quetiapine (n=20), haloperidol (n=1), paliperidone (n=5), chlorpromazine (n=1), blonanserin (n=1), escitalopram (n=7), sertraline (n=1), mirtazapine (n=2), duloxetine (n=1), venlafaxine (n=3), amitriptyline (n=1), trazodone (n=1), bupropion (n=1). Participants took an average of 1.91 (SD=1.02, range 0–5) different psychotropic drugs during ECT. The mean number of types of antipsychotics and antidepressants used were 1.53 (SD=0.74, range 0–3) and 0.37 (SD=0.76, range 0–4), respectively. Multivariate regression analyses showed positive correlations between initial ST and the total chlorpromazine-equivalent dose of antipsychotics (β = 0.363, p < 0.05). The total fluoxetine-equivalent dose of antidepressants was positively correlated to ΔST10th (β = 0.486, p < 0.05) and mean ΔSTlast (β = 0.472, p < 0.01).

**Discussion:**

Our study elucidated possible effects of psychotropic drugs on ST in patients undergoing ECT. We revealed that larger doses of antipsychotics are associated with higher initial ST, whereas higher doses of antidepressants are associated with stronger shifts of ST during the course of treatment. We believe that our findings provide a basis for creating safer and more efficient ECT protocols.

